# Intraoperative mechanical ventilation strategies in patients undergoing one-lung ventilation: a meta-analysis

**DOI:** 10.1186/s40064-016-2867-0

**Published:** 2016-08-03

**Authors:** Zhen Liu, Xiaowen Liu, Yuguang Huang, Jing Zhao

**Affiliations:** 1 Department of Anesthesiology, Peking Union Medical College Hospital, 1#Shuai fuyuan, Dongcheng District, Beijing, 100730 China; 2 Department of Anesthesiology, Plastic Surgery Hospital, Chinese Academy of Medical Sciences and Peking Union Medical College, 33# Shijingshan District, Beijing, 100144 China

**Keywords:** Protective ventilation, Conventional ventilation, One lung ventilation, Pressure-controlled ventilation, Volume-controlled ventilation

## Abstract

**Background:**

Postoperative pulmonary complications (PPCs), which are not uncommon in one-lung ventilation, are among the main causes of postoperative death after lung surgery. Intra-operative ventilation strategies can influence the incidence of PPCs. High tidal volume (*V*_T_) and increased airway pressure may lead to lung injury, while pressure-controlled ventilation and lung-protective strategies with low *V*_T_ may have protective effects against lung injury. In this meta-analysis, we aim to investigate the effects of different ventilation strategies, including pressure-controlled ventilation (PCV), volume-controlled ventilation (VCV), protective ventilation (PV) and conventional ventilation (CV), on PPCs in patients undergoing one-lung ventilation. We hypothesize that both PV with low *V*_T_ and PCV have protective effects against PPCs in one-lung ventilation.

**Methods:**

A systematic search (PubMed, EMBASE, the Cochrane Library, and Ovid MEDLINE; in May 2015) was performed for randomized trials comparing PCV with VCV or comparing PV with CV in one-lung ventilation. Methodological quality was evaluated using the Cochrane tool for risk. The primary outcome was the incidence of PPCs. The secondary outcomes included the length of hospital stay, intraoperative plateau airway pressure (P_plateau_), oxygen index (PaO_2_/FiO_2_) and mean arterial pressure (MAP).

**Results:**

In this meta-analysis, 11 studies (436 patients) comparing PCV with VCV and 11 studies (657 patients) comparing PV with CV were included. Compared to CV, PV decreased the incidence of PPCs (OR 0.29; 95 % CI 0.15–0.57; P < 0.01) and intraoperative P_plateau_ (MD −3.75; 95 % CI −5.74 to −1.76; P < 0.01) but had no significant influence on the length of hospital stay or MAP. Compared to VCV, PCV decreased intraoperative P_plateau_ (MD −1.46; 95 % CI −2.54 to −0.34; P = 0.01) but had no significant influence on PPCs, PaO_2_/FiO_2_ or MAP.

**Conclusions:**

PV with low *V*_T_ was associated with the reduced incidence of PPCs compared to CV. However, PCV and VCV had similar effects on the incidence of PPCs.

**Electronic supplementary material:**

The online version of this article (doi:10.1186/s40064-016-2867-0) contains supplementary material, which is available to authorized users.

## Background

One-lung ventilation, used to isolate and protect the lung, has been widely used in thoracic surgery. Nonphysiologic tidal volumes, loss of normal functional residual capacity and hyperperfusion in the ventilated lung during one-lung ventilation result in alveolar damage and inflammation response in the lung. These changes make patients susceptible to PPCs (Lohser and Slinger 2015). Various ventilation strategies, such as pressure-controlled ventilation (PCV), volume-controlled ventilation (VCV), conventional ventilation (CV) and protective ventilation (PV), are used in one-lung ventilation. The ideal ventilation strategy should minimize the risk of PPCs while also benefitting both gas exchange and pulmonary mechanics.

High tidal volume (*V*_T_) is associated with increased areas of overinflation but decreased areas of atelectasis at end-inspiration (Guldner et al. 2015). Protective ventilation with low *V*_T_ is thought to result in less ventilator-induced lung injury and has become a routine strategy in patients with ARDS (Petrucci and De Feo 2013). Recent studies have reported similar results in that low *V*_T_ prevents postoperative complications in surgical patients (Serpa Neto et al. 2015a, b). However, the effect of low *V*_T_ on patients undergoing one-lung ventilation remains unclear. In some studies, PV has been associated with a decreased oxygenation index and more dead space ventilation without decreases in the incidence of PPCs (Maslow et al. 2013; Jung et al. 2014; Blank et al. 2016; Neto et al. 2016). In other studies, PV was associated with a lower incidence of PPCs and satisfactory gas exchange (Schilling et al. 2005; Yang et al. 2011; Serpa Neto et al. 2015a, b). VCV and PCV are also used in one-lung ventilation. PCV may result in lower airway pressure and a more homogeneous distribution of the tidal volume; PCV also has less of an effect on cardiac function than VCV (Al Shehri et al. 2014). However, tidal volumes in PCV are highly variable (Della Rocca and Coccia 2013). The benefits of PCV in terms of oxygenation and protection against lung damage should be balanced.

This meta-analysis aims to investigate the association between ventilation strategies and PPCs; the length of hospital stay, intraoperative P_plateau_, PaO_2_/FiO_2_ and MAP were compared as the secondary outcomes. We hypothesize that PV with low *V*_T_ and PCV have protective effects on PPCs in one-lung ventilation.

## Methods

We used the Preferred Reporting Items for Systematic Review and Meta-analyses (PRISMA) recommended by the PRISMA working group (http://www.prisma-statement.org/) in this meta-analysis (Moher et al. 2009). This meta-analysis was registered on PROSPERO (Prospective Register of Ongoing Systematic Reviews, http://www.crd.york.ac.uk/prospero, Registration No. CRD42015022087).

### Eligibility criteria

We compared 2 types of interventions with 2 control groups. In the comparison of PCV and VCV, the intervention group was the PCV group and the control group was the VCV group. PCV was defined as ventilation under pressure control with or without PEEP in one-lung ventilation. VCV was defined as ventilation under volume control with or without PEEP in one-lung ventilation. In the comparison of PV and CV, the intervention group was the PV group and the control group was the CV group. According to previous studies, PV was defined as ventilation using low *V*_T_ (*V*_T_ ≤ 6 ml/kg predicted body weight) with or without PEEP and with or without alveolar recruitment strategies in one-lung ventilation. CV was defined as ventilation using *V*_T_ ≥ 7 ml/kg predicted body weight with or without PEEP and without recruitment maneuvers in one-lung ventilation (Lohser 2008; Della Rocca and Coccia 2013).

The included studies met the following criteria: randomized controlled trials of patients aged 18 years or older who were undergoing one-lung ventilation during a surgical procedure. Randomized clinical trials (RCTs) were excluded if they did not involve a surgical procedure, if they included patients undergoing cardiac surgery, if they included patients with cardiac diseases, sepsis or ARDS before surgery, if they were conference abstracts or if full-text articles could not be obtained, if they did not focus on the comparisons of different ventilation strategies in the dependent lung, if the intervention group and control group had different ventilation settings during two-lung ventilation (TLV), or if the RCTs did not report any outcomes mentioned above. Animal studies were also excluded.

The primary outcome of interest was the development of PPCs during follow up, defined as the development of atelectasis, lung infiltration, pneumonia or ARDS. The secondary outcomes included the length of hospital stay, intraoperative P_plateau_, PaO_2_/FiO_2_ and MAP. PaO_2_/FiO_2_ at 20–40 min in one-lung ventilation in randomized parallel studies was included in the analysis of PaO_2_/FiO_2_. Cross-over studies were not used to evaluate the effect of ventilation strategies on PPCs, the length of hospital stay or PaO_2_/FiO_2_.

### Information sources

We performed a literature search in PubMed, EMBASE, the Cochrane Library, and Ovid MEDLINE in May 2015. The last search was performed on May 14th, 2015.

### Search strategy

The terms ‘anesthesia’, ‘anaesthesia’, ‘surgery’, ‘surgical’, ‘operative’, ‘surgical operations’, ‘intra-operative care’, ‘postoperative care’, ‘preoperative care’, ‘perioperative care’, ‘one lung ventilation’, ‘single-lung ventilation’, ‘single-lung ventilations’, ‘lung separation techniques’, ‘lung separation technique’ were used in various combinations. The search was limited to clinical trials. The detailed search strategy is provided as Additional file 1.

### Study selection

Two reviewers (ZL, XWL) conducted the systematic search and independently reviewed the titles and abstracts of the studies. Only reports meeting the criteria listed above were included for data extraction, trial quality assessment and the analysis of results. Any disagreements among reviewers was resolved by discussion with a third author (JZ).

### Data collection process

Data were extracted independently by two reviewing authors (ZL, XWL). Authors of the original studies were contacted to provide additional information if necessary.

### Data items

The following information was extracted: study design (randomized parallel studies, randomized cross-over studies), number of patients, ventilation strategies, type of surgery, duration of one-lung ventilation, outcomes and preoperative FEV1 (% of predicted).

### Risk of bias

The Cochrane tool for risk of bias was used to assess the risk of bias for all studies (Higgins et al. 2011). The risk of bias for random sequence generation, allocation concealment, blinding of participants and personnel, blinding of outcome assessment, incomplete outcome data, selective reporting and others was evaluated and classified as “low”, “high”, or “unclear” risk. We used funnel plots to assess reporting bias, and these plots are available in the Additional file 1. The risk of bias evaluation was conducted independently by two authors (ZL, XWL).

### Synthesis of results

Review-Manager software (RevMan, version 5.3; The Cochrane Collaboration, Oxford, UK) was used to conduct the data analysis. For binary outcomes, PPCs were summarized using odds ratios and their 95 % confidence intervals (CIs). Mean difference and 95 % CI were reported for continuous outcomes. We used I^2^ to estimate heterogeneity within the studies (Higgins et al. 2003). A fixed-effect model was used to analyze the data. If I^2^ was greater than 50 %, we utilized the random-effects model.

### Additional analysis

When comparing the clinical effect of PCV with VCV, we performed subgroup analyses to determine if outcomes could be influenced by the setting of *V*_T_ or the type of PCV. The setting of *V*_T_ was divided into *V*_T_ ≤ 6 ml/kg and *V*_T_ ≥ 7 ml/kg predicted body weight. The type of PCV was divided into conventional pressure-controlled ventilation and PCV-VG. Subgroup analysis was performed only when there were no less than 3 studies providing information for one outcome. If I^2^ > 50 %, we performed a sensitivity analysis by removing trials and reanalyzing the remaining studies.

### Quality assessment

A Measure Tool to Assess Systematic Reviews (AMSTAR) was applied to assess the methodological quality. Grading of Recommendations Assessment, Development and Evaluation (GRADE) system was used to assess the evidence quality.

## Results

### Study selection

**Fig. 1:**
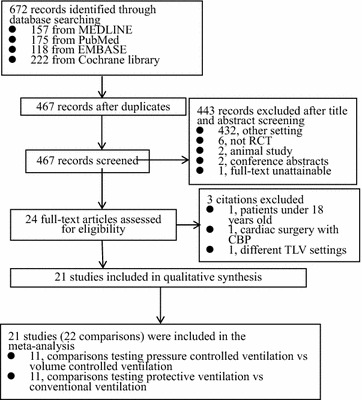
Study flow diagram

A total of 467 studies were screened and assessed for eligibility. Of these, 446 records were excluded for a variety of reasons as shown in Fig. 1. The remaining 21 randomized controlled trials (reporting on 22 comparisons) involving 1083 one-lung ventilation patients undergoing non-cardiac surgery were included in this meta-analysis. Eleven studies including 436 patients compared PCV with VCV, and 11 studies including 657 patients compared PV with CV. Data shown in the histogram were collected by contacting the corresponding authors by e-mail for one trial (Jung et al. 2014).

### Study characteristics

Eleven of the 21 studies included in this meta-analysis were randomized parallel studies and 10 studies were randomized cross-over studies. The study sample sizes ranged from 18 to 120 patients. The trials involved both open thoracic surgery and video-assisted thoracic surgery. The duration of one-lung ventilation ranged from 73.4 to 109 min. The characteristics and risk of bias in each study are shown in Tables 1 and 2, respectively.

**Table 1:** Characteristics of included studies

Author	Year	No. of patients	Study intervention	Details of *V*_T_ (ml/kg) and PEEP (cmH_2_O)	Age (year), mean (SD)	Type of surgery	Duration of one-lung ventilation in each mode (min), mean (SD)	Outcomes	Time point of measurement of outcomes	Preoperative FEV1 (% of predicted) (SD)
*Randomized parallel study*										
Hu et al.	2014	(1) 15 (2) 15	(1) PCV-VG (2) VCV	(1) *V*_T_ = 7 PEEP = 0 (2) *V*_T_ = 7 PEEP = 0	(1) 61 (6) (2) 62 (7)	VATS	(1) 147(22) (2) 154(52)	Respiratory parameters, gas exchange, hemodynamics	15 and 60 min after OLV	Not reported
Qutubet al.	2014	(1) 13 (2) 13 (3) 13	(1) PV 4 (2) PV 6 (3) CV	(1) *V*_T_ = 4 PEEP = 5 (2) *V*_T_ = 6 PEEP = 5 (3) *V*_T_ = 8 PEEP = 5	(1) 42 (32–54) (2) 39.5 (31–51) (3) 36 (29–48)	VATS	Not reported	Extravascular lung water content index, respiratory parameters, gas exchange, clinical outcomes	15, 45 min after OLV; 48 h, 30d post operation	(1) 87.1 (3.0) (2) 88.4 (2.9) (3) 85.8 (4.1)
Jung et al.	2014	(1) 30 (2) 30	(1) PV (2) CV	(1) *V*_T_ = 6 PEEP = 8 (2) *V*_T_ = 10 PEEP = 0	(1) 35.2 (10.2) (2) 36.3 (9.5)	VATS	Not reported	Respiratory parameters	5, 15, 30, 45 min after OLV	Not reported
Shen et al.	2013	(1) 53 (2) 48	(1) PV (2) CV	(1) *V*_T_ = 5 PEEP = 5 (2) *V*_T_ = 8 PEEP = 0	(1) 60.5 (7.3) (2) 57.2 (9.1)	MIE	(1) 72.2 (23.6) (2) 75.0 (18.8)	Clinical outcomes, gas exchange, cytokines expression	18 h post operation, 30d post operation	(1) 92.8 (14.6) (2) 87.1 (16.9)
Maslow et al.	2013	(1) 16 (2) 16	(1) PV (2) CV	(1) *V*_T_ = 5 PEEP = 5 (2) *V*_T_ = 10 PEEP = 0	(1) 62 (14.4) (2) 69.6 (12.9)	Thoracic surgery	(1) 42 (8.3) (2) 46 (9.5)	Respiratory parameters, gas exchange, hemodynamics, clinical outcomes	5, 10, 15, 20, 30 min after OLV	(1) 85.8 (21.7) (2) 75.4 (16.4)
Ye and Li	2011	(1) 10 (2) 10 (3) 10	(1) PCV (2) VCV (3) PV	(1) *V*_T_ = 8 PEEP = 0 (2) *V*_T_ = 8 PEEP = 0 (3) *V*_T_ = 6 PEEP = 5	20–65	Thoracic surgery	Not reported	Respiratory parameters, gas exchange	20, 45 and 70 min after OLV	Not reported
Yang et al.	2011	(1) 50 (2) 50	(1) PV (2) CV	(1) *V*_T_ = 6 PEEP = 5 (2) *V*_T_ = 10 PEEP = 0	(1) 58 (12) (2) 60 (10)	Thoracic surgery	(1) 120 (41) (2) 126 (53)	Clinical outcomes, gas exchange, hemodynamics	15, 60 min after OLV; 2, 72 h post operation	(1) 105 (9) (2) 104 (17)
Boules and Ghobrial	2011	(1) 18 (2) 19	(1) PCV-VG (2) VCV	(1) *V*_T_ = 6 PEEP = 0 (2) *V*_T_ = 6 PEEP = 0	(1) 33.4 (6.4) (2) 34.7 (7.6)	Thoracic surgery	(1) 88.7 (42.1) (2) 75.6 (34.7)	Respiratory parameters, gas exchange, hemodynamics, clinical outcomes	30 min after OLV;72 h post operation	(1) 73.4 (11.7) (2) 74.3 (12.5)
Pardos et al.	2009	(1) 55 (2) 55	(1) PCV +PEEP (2) VCV + PEEP	(1) *V*_T_ = 8 PEEP = 0; 20 min after OLV PEEP = 5 (2) *V*_T_ = 8 PEEP = 0; 20 min after OLV PEEP = 5	(1) 59.5 (13) (2) 63.9 (11)	Thoracic surgery	Not reported	Respiratory parameters, gas exchange, clinical outcomes	20, 30 and 40 min after OLV; 24 h post operation; 30d post operation	(1) 91.2 (24) (2) 87.9 (21)
Lin et al.	2008	(1) 20 (2) 20	(1) PV (2) CV	(1) *V*_T_ = 5–6 PEEP = 3–5 (2) *V*_T_ = 10 PEEP unclear	(1) 55 (2) 54	Thoracic surgery	Not reported	Cytokines expression, respiratory parameters, gas exchange	120 min after OLV, 24 h post operation	Not reported
Michelet et al.	2006	(1) 26 (2) 26	(1) PV (2) CV	(1) *V*_T_ = 5 PEEP = 5 (2) *V*_T_ = 9 PEEP = 0	(1) 61 (10) (2) 60 (8.5)	Thoracic surgery	(1) 85 (29) (2) 89 (29)	Cytokines expression, respiratory parameters, gas exchange, clinical outcomes	15 min after OLV; at the end of OLV; 1,18 h post operation	(1) 93 (19) (2) 96 (18)
*Randomized cross*-*over study*										
Song et al.	2014	27	(1) PCV-VG (2) VCV	(1) *V*_T_ = 8 PEEP = 0 (2) *V*_T_ = 8 PEEP = 0	63.6 (9.7)	Thoracic surgery	(1) 30 (2) 30	Respiratory parameters, gas exchange, hemodynamics	30 min after OLV in each mode	107.3 (33.1)
Pu et al.	2014	20	(1) PCV-VG (2) VCV	(1) *V*_T_ = 8–10 PEEP unclear (2) *V*_T_ = 8–10 PEEP unclear	59.8 (unclear)	Thoracic surgery	(1) 30 (2) 30	Respiratory parameters, gas exchange, hemodynamics	30 min after OLV in each mode	Not reported
Al Shehri et al.	2014	28	(1) PCV (2)VCV	(1) *V*_T_ = 6 PEEP = 5 (2) *V*_T_ = 6 PEEP = 5	(1) 37.4 (11.51) (2) 39.1 (13.93)	Thoracic surgery	(1) 30 (2) 30	Right ventricular function, gas exchange, hemodynamics	30 min after OLV in each mode	84.5 (10.8)
Végh et al.	2013	100	(1) PV (2) CV	(1) *V*_T_ = 5 PEEP = 5 (2) *V*_T_ = 10 PEEP = 0	(1) 64 (12) (2) 63 (12)	Thoracic surgery	(1) 30 (2) 30	Respiratory parameters, gas exchange, hemodynamics	30 min after OLV in each mode	91.5 (14.0)
Roze et al.	2012	82	(1) PV (2) CV	(1) *V*_T_ = 5 PEEP = 9 (1) (2) *V*_T_ = 8 PEEP = 5	(1) 62 (10) (2) 60 (10)	Thoracic surgery	(1) 10 (2) 10	Respiratory parameters, Gas exchange, hemodynamics	10 min after OLV in each mode	Not reported
Sungur Ulke et al.	2011	31	(1) PV (2) CV	(1) *V*_T_ = 6 PEEP = 5 (2) *V*_T_ = 8 PEEP = 0	58.3 (7.2)	Thoracic surgery	(1) 20 (2) 20	Respiratory parameters, gas exchange, hemodynamics	20 min after OLV in each mode	75 (14.7)
Montes et al.	2010	41	(1) PCV (2)VCV	(1) *V*_T_ = 6 PEEP = 5 (2) *V*_T_ = 6 PEEP = 5	(1) 59.1 (16) (2) 56.1 (17)	Thoracic surgery	(1) 30 (2) 30	Respiratory parameters, gas exchange	30 min after OLV in each mode	91.2 (19.3)
Choi et al.	2009	18	(1) PCV (2) VCV	(1) *V*_T_ = 8 PEEP = 0 (2) *V*_T_ = 9 PEEP = 0	61.4 (10.3)	Robot-assisted esophagectomy	(1) 30 (2) 30	Respiratory parameters, gas exchange, hemodynamics	30 min after OLV in each mode	109.1 (21.2)
Unzueta et al.	2007	57	(1) PCV (2) VCV	(1) *V*_T_ = 9 PEEP = 0 (2) *V*_T_ = 9 PEEP = 0	(1) 58.25 (15.15) (2) 54.75 (13.91)	Thoracic surgery	(1) 30 (2) 30	Respiratory parameters, gas exchange	30 min after OLV in each mode	82.2 (17.5)
Tugrul et al.	1997	48	(1) PCV (2) VCV	(1) *V*_T_ = 10 PEEP unclear (2) *V*_T_ = 10 PEEP unclear	56.4	Thoracic surgery	(1) 30 (2) 30	Respiratory parameters, gas exchange, hemodynamics	30 min after OLV in each mode	76.8 (14)

*ARS* alveolar recruitment strategy, *CV* conventional ventilation, *MIE* minimally invasive esophagectomy, *PCV* pressure-controlled ventilation, *PCV*-*VG* volume guaranteed pressure-controlled ventilation, *PV* protective ventilation, *VATS* video-assisted thoracoscopic surgery, *VCV* volume-controlled ventilation

**Table 2:** Risk of bias in included studies

Author	Year	Random sequence generation	Allocation concealment	Blinding of participants and personnel	Blinding of outcome assessment	Incomplete outcome data	Selective reporting	Other bias
*Randomized parallel study*								
Hu et al.	2014	Low	High	Low	Low	Unclear	Unclear	Low
Qutub et al.	2014	Unclear	Unclear	Low	Low	Low	Low	Low
Jung et al.	2014	Low	High	Low	Low	Unclear	Unclear	Low
Shen et al.	2013	Low	Unclear	Low	Unclear	Low	High	Low
Maslow et al.	2013	Unclear	Unclear	Low	Unclear	Low	Unclear	Low
Yang et al.	2011	Low	Low	Low	Low	Low	Unclear	Low
Ye and Li	2011	Unclear	Unclear	Low	Low	Unclear	Unclear	Low
Boules and Ghobrial	2011	Low	Unclear	Low	Unclear	Unclear	Unclear	Low
Pardos et al.	2009	Unclear	High	Low	Unclear	Unclear	Unclear	Low
Lin et al.	2008	Unclear	Unclear	Low	Low	Unclear	Unclear	Low
Michelet et al.	2006	Low	Low	Low	Unclear	Low	Unclear	High
*Randomized cross*-*over study*								
Song et al.	2014	Low	Unclear	Low	Low	Unclear	Unclear	Low
Pu et al.	2014	Unclear	Unclear	Low	Low	Unclear	Unclear	Low
Al Shehri et al.	2014	Low	Low	Low	Low	Unclear	Low	High
Végh et al.	2013	Low	Unclear	Low	Low	Unclear	Low	Low
Roze et al.	2012	Low	Unclear	Low	Low	Low	Low	Low
Sungur Ulke et al.	2011	Low	Unclear	Low	Low	Unclear	Unclear	Low
Montes et al.	2010	Low	Unclear	Low	Low	Low	Unclear	Low
Choi et al.	2009	Low	Unclear	Low	Low	Low	Unclear	Low
Unzueta et al.	2007	Low	Unclear	Low	Low	Low	Unclear	Low
Tugrul et al.	1997	Unclear	Unclear	Low	Low	Unclear	Unclear	Low

### Results of individual studies and synthesis of results

#### Postoperative pulmonary complications

**Fig. 2:**
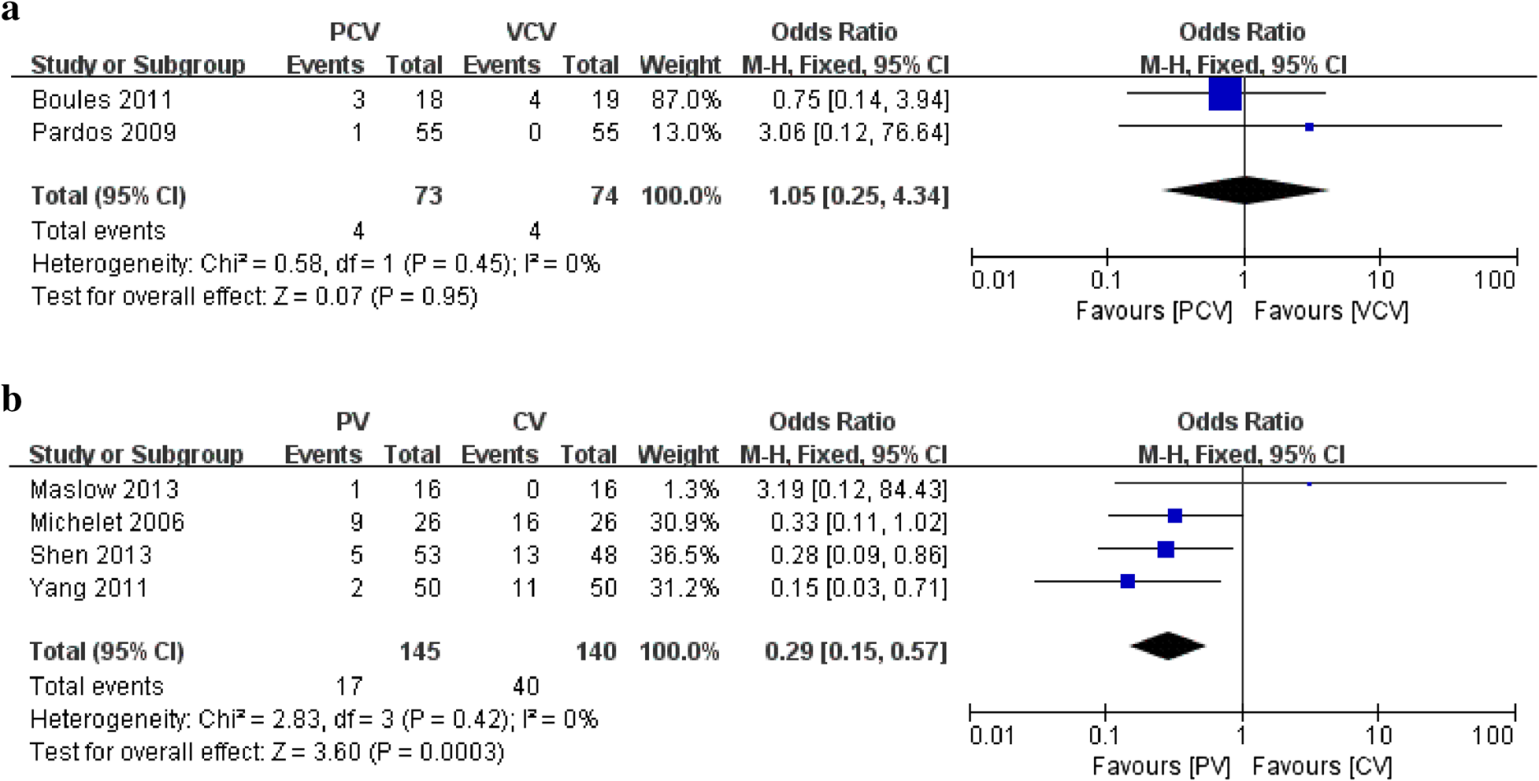
Effect of ventilation strategies on postoperative pulmonary complications. **a** PCV versus VCV; **b** PV versus CV

Two of the 11 studies including 147 patients comparing PCV with VCV reported PPCs as an outcome (Pardos et al. 2009; Boules and Ghobrial 2011). Both studies found that PCV did not have any advantages over VCV in terms of decreased incidence of PPCs (OR 1.05; 95 % CI 0.25–4.34; I^2^ = 0; P = 0.95) (Fig. 2a).

Four of the 12 studies including 285 patients comparing PV with CV reported PPCs as an outcome (Michelet et al. 2006; Yang et al. 2011; Maslow et al. 2013; Shen et al. 2013). PV showed a protective effect over CV on respiratory complications after one-lung ventilation (OR 0.29; 95 % CI 0.15–0.57; I^2^ = 0; P < 0.01) (Fig. 2b).

#### Length of hospital stay

**Fig. 3:**
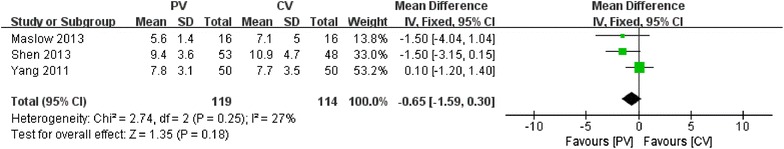
Effect of ventilation strategies on length of hospital stay (days)

Four studies including 272 patients comparing PV with CV reported the length of hospital stay as an outcome (Yang et al. 2011; Maslow et al. 2013; Shen et al. 2013; Qutub et al. 2014). No advantages in terms of the length of hospital stay were found in the PV group (MD −0.65; 95 % CI −1.59 to 0.30; I^2^ = 27 %; P = 0.18) (Fig. 3).

#### Plateau airway pressure

**Fig. 4:**
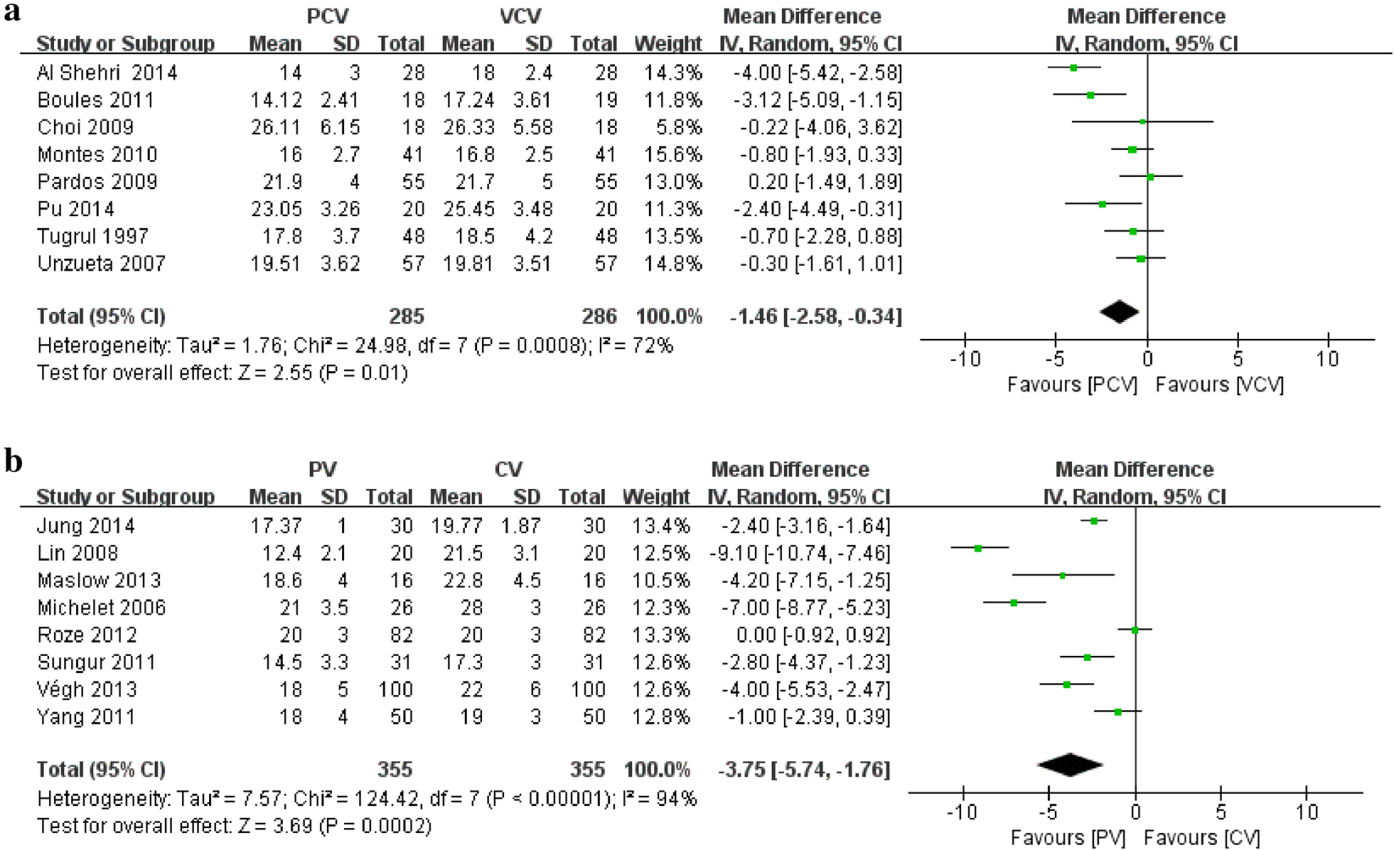
Effect of ventilation strategies on plateau airway pressure. **a** PCV versus VCV; **b** PV versus CV

Eight of the 11 studies including 359 patients comparing PCV with VCV reported P_plateau_ as an outcome (Tugrul et al. 1997; Unzueta et al. 2007; Choi et al. 2009; Pardos et al. 2009; Montes et al. 2010; Boules and Ghobrial 2011; Al Shehri et al. 2014; Pu et al. 2014). PCV showed decreased P_plateau_ compared to VCV (MD −1.46; 95 % CI −2.58 to −0.34; I^2^ = 72 %; P = 0.01) (Fig. 4a).

Eight of the 12 studies including 497 patients comparing PV with CV reported P_plateau_ as an outcome (Michelet et al. 2006; Lin et al. 2008; Sungur Ulke et al. 2011; Yang et al. 2011; Roze et al. 2012; Maslow et al. 2013; Végh et al. 2013; Jung et al. 2014). PV decreased P_plateau_ compared to CV (MD −3.57; 95 % CI −5.74 to −1.76; I^2^ = 94 %; P < 0.01) (Fig. 4b).

#### PaO_2_/FiO_2_

**Fig. 5:**
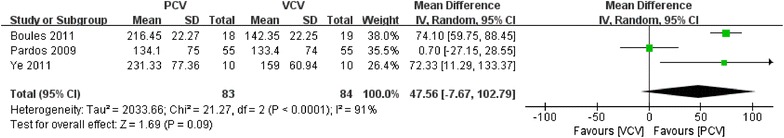
Effect of ventilation strategies on PaO_2_/FiO_2_

Three randomized parallel trials including 167 patients comparing PCV and VCV reported PaO_2_/FiO_2_ at 20–30 min after OLV as an outcome (Pardos et al. 2009; Boules and Ghobrial 2011; Ye and Li 2011). No differences in PaO_2_/FiO_2_ were found in those 2 groups (MD 47.56; 95 % CI −7.67 to 102.79; I^2^ = 91 %; P = 0.09) (Fig. 5).

#### Mean arterial pressure

**Fig. 6:**
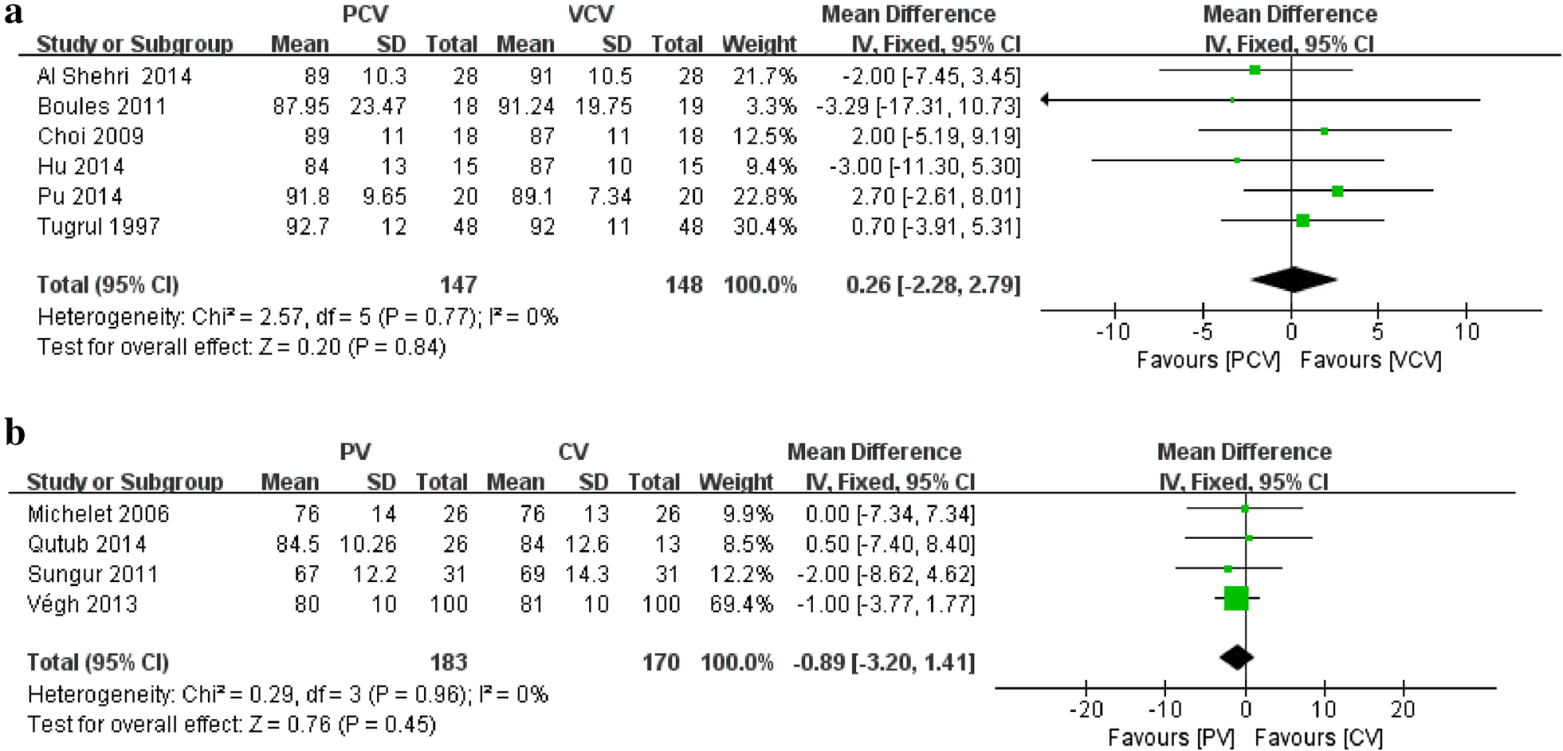
Effect of ventilation strategies on mean arterial pressure. **a** PCV versus VCV; **b** PV versus CV

Six of the 11 studies including 181 patients comparing PCV and VCV reported MAP as an outcome (Tugrul et al. 1997; Choi et al. 2009; Boules and Ghobrial 2011; Al Shehri et al. 2014; Hu et al. 2014; Pu et al. 2014). No differences in MAP were found in those 2 groups (MD 0.26; 95 % CI −2.28–2.79; I^2^ = 0; P = 0.84) (Fig. 6a).

Four of the 12 studies including 222 patients comparing PV and CV reported MAP as an outcome (Michelet et al. 2006; Sungur Ulke et al. 2011; Végh et al. 2013; Qutub et al. 2014). No differences in MAP were found in those 2 groups (MD −0.89; 95 % CI −3.20 to 1.41; I^2^ = 0; P = 0.45) (Fig. 6b).

#### Subgroup analysis

Subgroup analysis regarding the volume of *V*_T_ showed a decrease in P_plateau_ (MD −2.58; 95 % CI −4.74 to −0.43; I^2^ = 85 %; P = 0.02) in patients undergoing PCV with *V*_T_ 6 ml/kg predicted body weight compared to VCV. In the groups with *V*_T_ ≥ 7 ml/kg, no significant benefit was found in patients undergoing PCV compared to VCV (MD −0.58; 95 % CI −1.37–0.20) (Table 3).

**Table 3:** Subgroup analyses of patients undergoing one-lung ventilation with PCV and VCV

	Volume of *V*_T_		Type of PCV	
	≤6 ml/kg	≥7 ml/kg	Traditional PCV	PCV-VG
Plateau airway pressure [MD (95 % CI)]	−2.58 (−4.74, −0.43)	−0.58 (−1.37, 0.20)	−1.06 (−2.37, 0.24)	−2.78 (−4.21, −1.35)
Mean arterial pressure [MD (95 % CI)]	−2.17 (−7.25, 2.91)	1.06 (−1.87, 3.99)	0.04 (−3.12, 3.20)	0.64 (−3.62, 4.91)

Subgroup analysis on the type of PCV showed decreases in P_plateau_ (MD −2.78; 95 % CI −4.21 to −1.35; I^2^ = 0 %; P < 0.01) in patients undergoing volume guaranteed pressure-controlled ventilation (PCV-VG) compared to VCV, while traditional PCV showed no significant benefits in P_plateau_ (MD −1.06; 95 % CI −2.37–0.24; I^2^ = 75 %; P = 0.11). With respect to MAP, no significant differences or heterogeneity were found in the subgroup analysis.

Sensitivity analyses of P_plateau_ and PaO_2_/FiO_2_ were also performed. When comparing PCV with VCV, heterogeneity in P_plateau_ could be resolved by excluding the study by Al Shehri et al. (2014) (MD −0.89; 95 % CI −1.50 to −0.28; I^2^ = 37 %; P < 0.01). This change had no effect on the final result. Heterogeneity in PaO_2_/FiO_2_ could be resolved by excluding the study by Pardos et al. (Pardos et al. 2009) (MD 74.01; 95 % CI 60.04–87.98; I^2^ = 0 %; P < 0.01). This change affected the final result and showed PCV benefited PaO_2_/FiO_2_ in comparison with VCV. In the comparison of PV with CV on P_plateau_, heterogeneity and the final result could not be resolved by the exclusion of any study involved in this meta-analysis.

## Discussion

This meta-analysis suggests that PV but not PCV can decrease the incidence of PPCs. Although both PV and PCV can decrease the P_plateau_, subgroup analyses show that PCV-VG (but not traditional PCV) can decrease P_plateau_. Currently available data are insufficient to identify differences between PV and CV or PCV and VCV on the length of hospital stay, PaO_2_/FiO_2_ or MAP.

Our result suggesting that PV with low *V*_T_ can protect surgical patients from PPCs is consistent with recently published studies (Hemmes et al. 2015, Serpa Neto et al. 2015a, b). However, the definition of PV in these studies is ventilation with *V*_T_ ≤ 8 ml/kg, and they also include all surgical patients under general anesthesia (Hemmes et al. 2015). The definition of *V*_T_ and the conclusions from these studies might not be suitable in one-lung ventilation. Our results suggest that PV with *V*_T_ ≤ 6 ml/kg can benefit surgical patients in one-lung ventilation. A high quality retrospective study published recently found that low *V*_T_ does not prevent PPCs, which contradicts our results (Blank et al. 2016). In this retrospective study, fewer than half (47 %) of the patients received PEEP ≥ 5 cmH_2_O (Blank et al. 2016). Atelectasis should be considered in all general anesthetized patients. And it is of great importance to avoiding the occurrence of atelectasis during OLV (Lohser and Slinger 2015). Low *V*_T_ with low PEEP can cause increased amounts of atelectasis (Guldner et al. 2015). Only one study with a sample size of 40 patients used PV with PEEP ≤ 5 cmH_2_O (Lin et al. 2008). In this study performed by Lin et al., PPCs were not included in the outcomes (Lin et al. 2008). To achieve a protective effect on PPCs, PEEP ≥ 5 cmH_2_O may be necessary when PV is used in surgical patients undergoing one-lung ventilation.

P_plateau_ is part of the driving pressure and contributes to ventilator-induced lung injury (Neto et al. 2016). Our results suggest that PV has lower P_plateau_ compared to CV, which might explain the mechanism of decreased PPCs in the PV group. Although PCV can also decrease the P_plateau_ compared to VCV, current data are insufficient to identify any difference between PCV and VCV on PPCs. It should be noted that P_plateau_ in PV is lower than P_plateau_ in PCV on average (Choi et al. 2009; Sungur Ulke et al. 2011). Differences in P_plateau_ may be caused by the differences in *V*_T_. The *V*_T_ in PCV is usually 8 ml/kg or higher, while the *V*_T_ in PV is no more than 6 ml/kg (Michelet et al. 2006; Jung et al. 2014; Pu et al. 2014). PCV-VG is a novel mode of ventilation which has been used in recent years. Although present data suggest that PCV-VG can decrease P_plateau_, more studies are still needed.

A combination of low *V*_T_ and PEEP is generally used in PV. PEEP can cause cardiac compromise, which can be reflected by MAP. Low *V*_T_ may induce hypoxemia. In this meta-analysis, the effects of different ventilation strategies on PaO_2_/FiO_2_ and MAP were compared. Decreasing alveolar oxygen tension could induce HPV and resulted in the redistribution of pulmonary blood flow (Moudgil et al. 2005). HPV had a rapid-onset phase and a delayed phase in response to alveolar hypoxia. The rapid-onset phase reached a plateau at 20–40 min. The delayed phase begins at 40 min and takes more than 2 h to reverse (Lumb and Slinger 2015). PaO_2_/FiO_2_ can be affected by HPV. Because most thoracic surgery can be completed in 2 h, only the results of PaO_2_/FiO_2_ at 20 to 40 min after one-lung ventilation are compared in this meta-analysis. Our results suggest that there is no difference between PV and CV or PCV and VCV on PaO_2_/FiO_2_ and MAP. This result is consistent with clinical studies published previously (Boules and Ghobrial 2011; Qutub et al. 2014).

The studies included in this meta-analysis are all RCTs and the overall quality of their reporting is good. Random sequence generation and allocation concealment are utilized in most studies. This meta-analysis is of high methodological quality assessed by AMSTAR. However, limited by the number of patients, the overall strength of the evidence provided by this meta-analysis is moderate (Additional file 2).

This meta-analysis has some limitations. First, PPCs include a combination of various lung injuries after surgery. The incidence of atelectasis, volutrauma, barotrauma and ARDS may not be the same with different ventilation strategies. However, this definition of PPCs is thought to be a stronger outcome than a single complication analysis (Hemmes et al. 2015). Second, the surgical procedure is one of the factors that could influence the incidence of PPCs (Licker et al. 2003). Some studies in this meta-analysis did not report the exact surgical procedure and currently available data cannot meet the criteria of subgroup analysis on surgical procedure. However, the differences in surgical procedure can be minimized by randomization. All studies included in this meta-analysis were of randomized design. Third, the length of hospital stay was a combination of ICU days and non-ICU days in many studies included in the meta-analysis. Therefore, the results on the length of hospital stay should be interpreted with caution.

## Conclusions

This meta-analysis suggests that protective ventilation with low *V*_T_ protects surgical patients against PPCs in one-lung ventilation. Further trials are needed to define the role of PCV in preventing PPCs in one-lung ventilation.

## Additional files

10.1186/s40064-016-2867-0 Subgroup analysis, publication bias, GRADE system assessment and search strategies of this meta-analysis.
10.1186/s40064-016-2867-0 PRISMA Checklist of this meta-analysis.

## Abbreviations

ARDS: respiratory distress syndrome
CV: conventional ventilation
HPV: hypoxic pulmonary vasoconstriction
MAP: mean arterial pressure
PaO_2_/FiO_2_: oxygen index
PCV: pressure-controlled ventilation
PCV-VG: volume guaranteed pressure-controlled ventilation
PEEP: positive end expiratory pressure
P_plateau_: intraoperative plateau airway pressure
PV: protective ventilation
VCV: volume-controlled ventilation
*V*_T_: tidal volume
