# Supplementary material for: Intraoperative mechanical ventilation strategies in patients undergoing one-lung ventilation: a meta-analysis
Source: Springerplus. 2016 Aug 3;5(1):1251. doi: 10.1186/s40064-016-2867-0 (PMC4972804; doi:10.1186/s40064-016-2867-0)
Supplement: Supplementary file 1 — 10.1186/s40064-016-2867-0 Subgroup analysis, publication bias, GRADE system assessment and search strategies of this meta-analysis. [file 40064_2016_2867_MOESM1_ESM.doc]

**Supplementary Material**

**Supplementary Figures**

Figure S1 Pooled estimates of plateau airway pressure according to volume of *V*_T_ comparing PCV versus VCV.


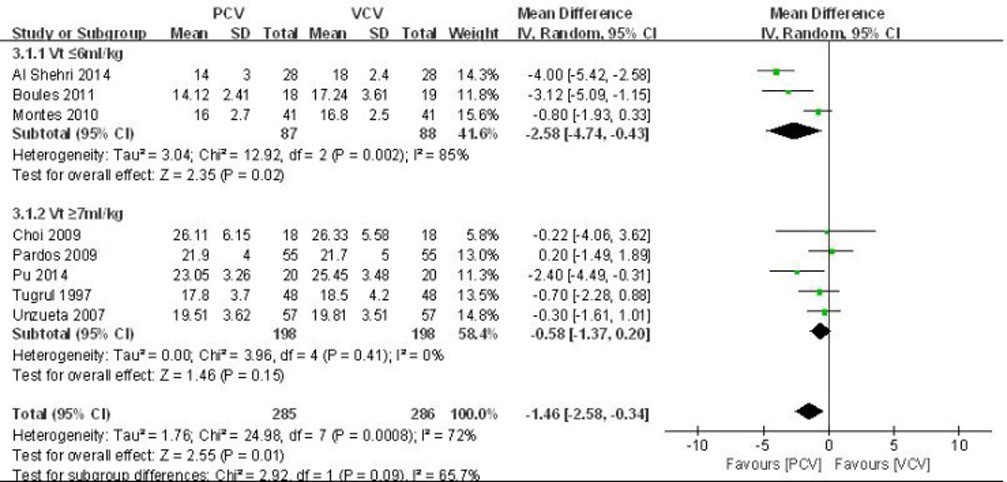


Figure S2 Pooled estimates of MAP according to volume of *V*_T_ comparing PCV versus VCV.


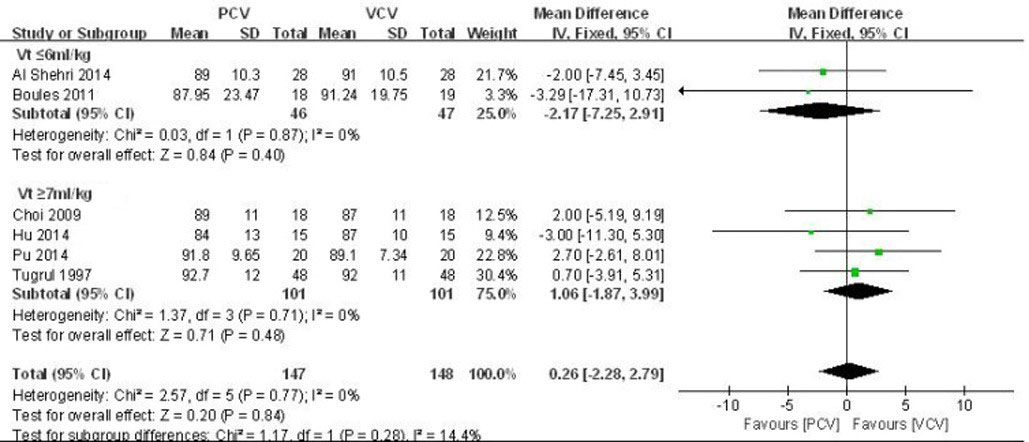


Figure S3 Pooled estimates of plateau airway pressure according to type of PCV comparing PCV versus VCV


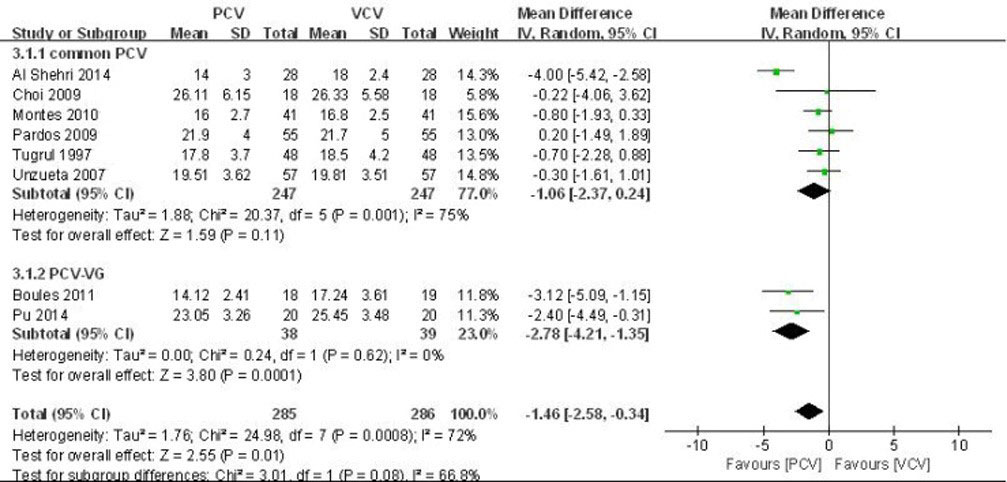


Figure S4 Pooled estimates of MAP according to type of PCV comparing PCV versus VCV


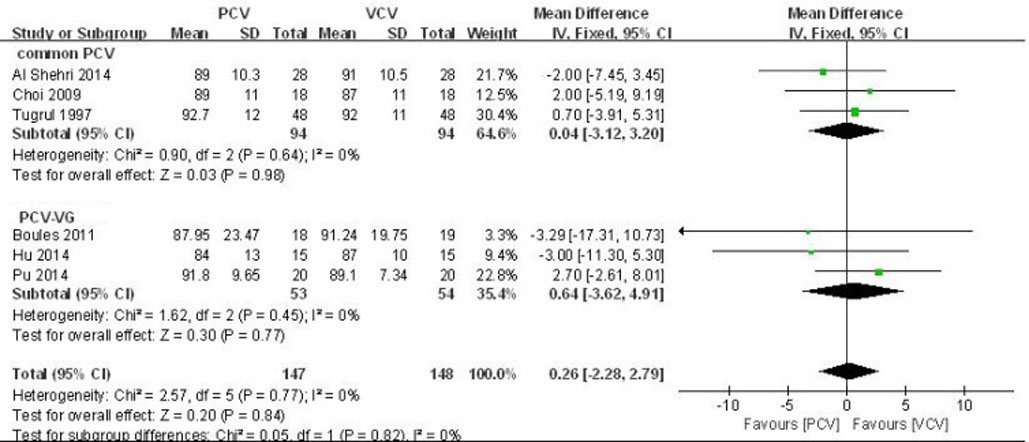


**Funnel plots**

1. Postoperative respiratory complications.


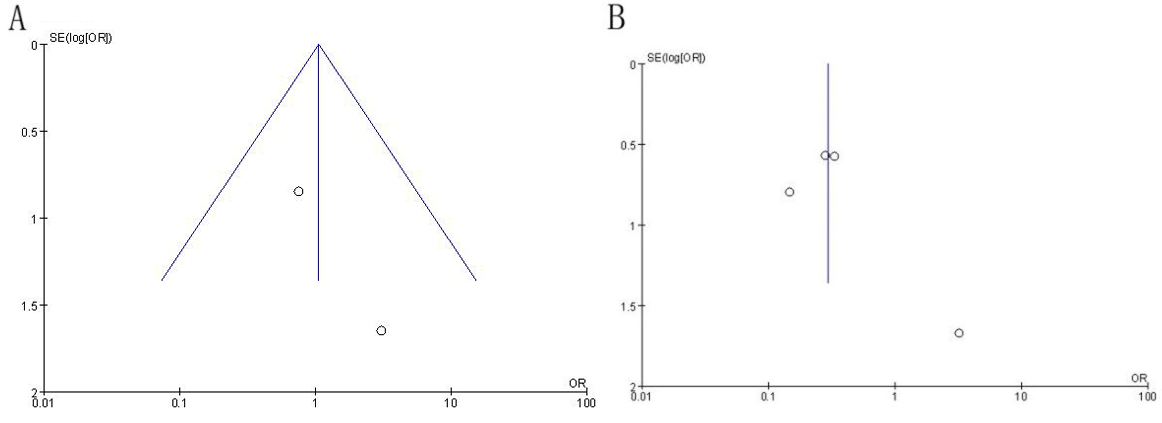


1. pressure-controlled ventilation versus volume-controlled ventilation (B) protective ventilation versus conventional ventilation
2. Length of hospital stay (protective ventilation versus conventional ventilation)


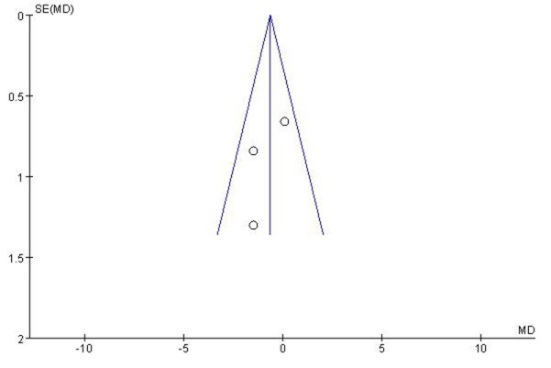


1. Plateau airway pressure


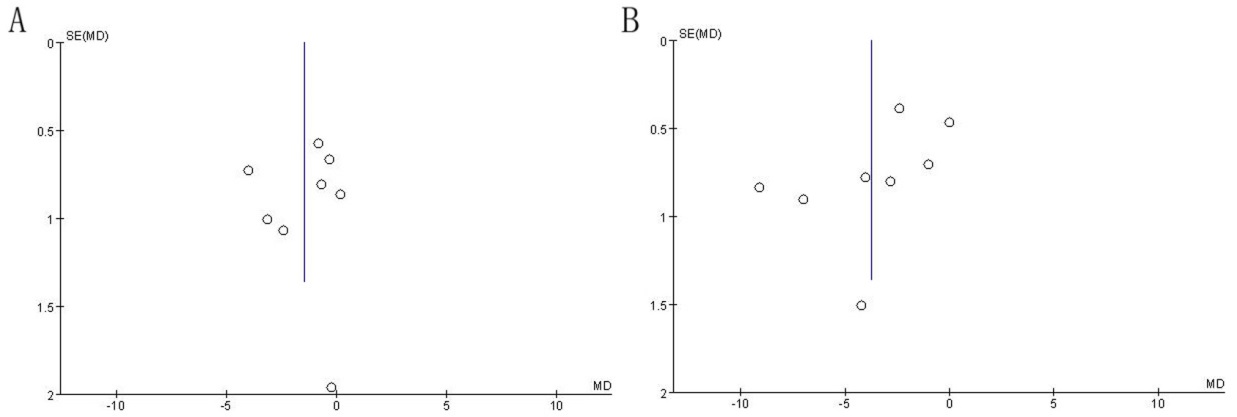


1. pressure-controlled ventilation versus volume-controlled ventilation (B) protective ventilation versus conventional ventilation
2. PaO_2_/FiO_2_


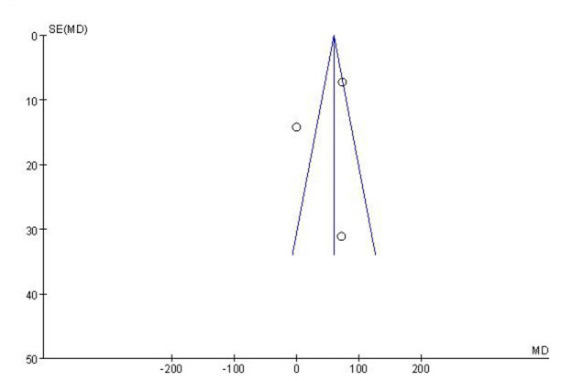


1. Mean arterial pressure


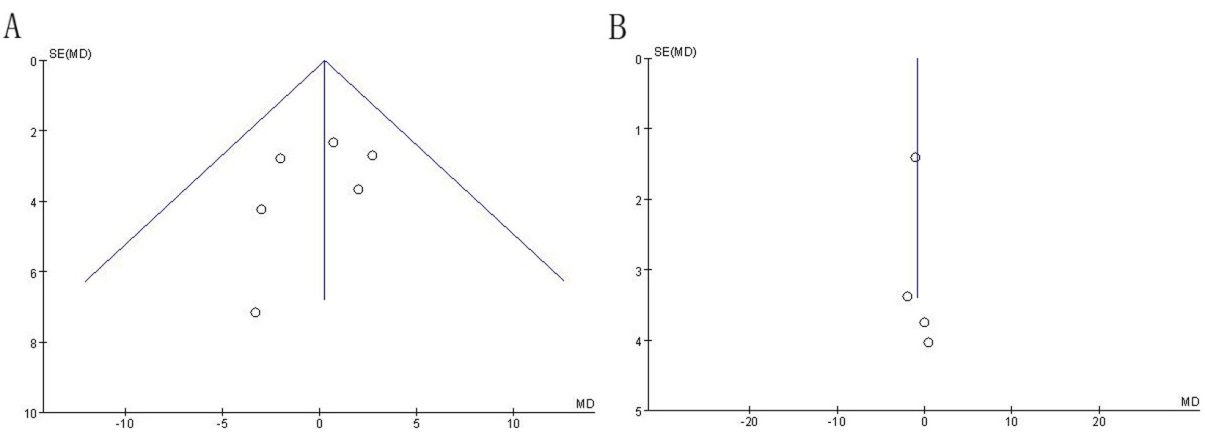


1. pressure-controlled ventilation versus volume-controlled ventilation (B) protective ventilation versus conventional ventilation

**GRADE system assessment**

1. Postoperative respiratory complications.


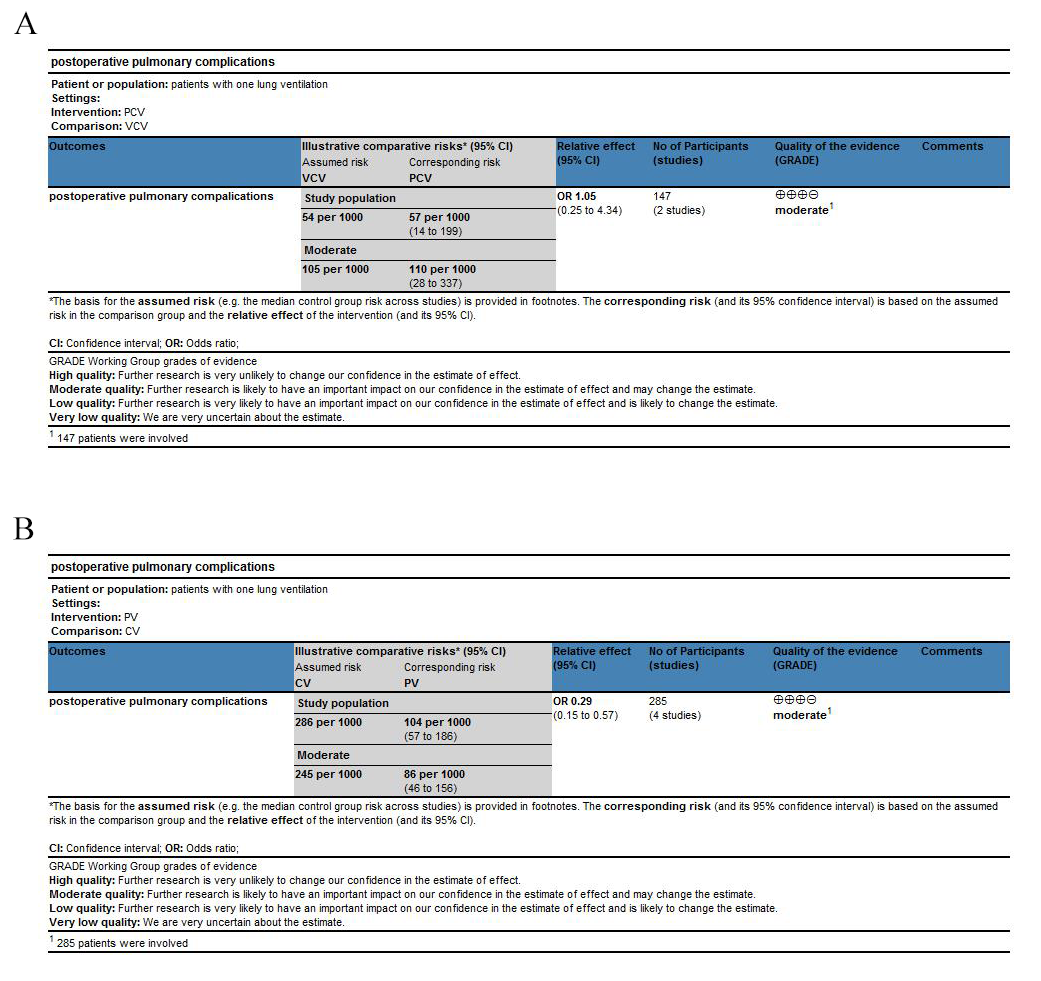


1. pressure-controlled ventilation versus volume-controlled ventilation (B) protective ventilation versus conventional ventilation
2. Length of hospital stay (protective ventilation versus conventional ventilation)


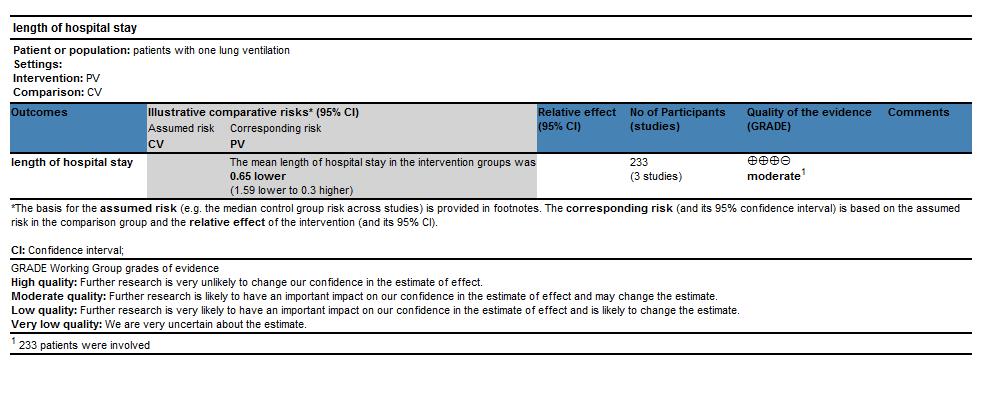


1. Plateau airway pressure


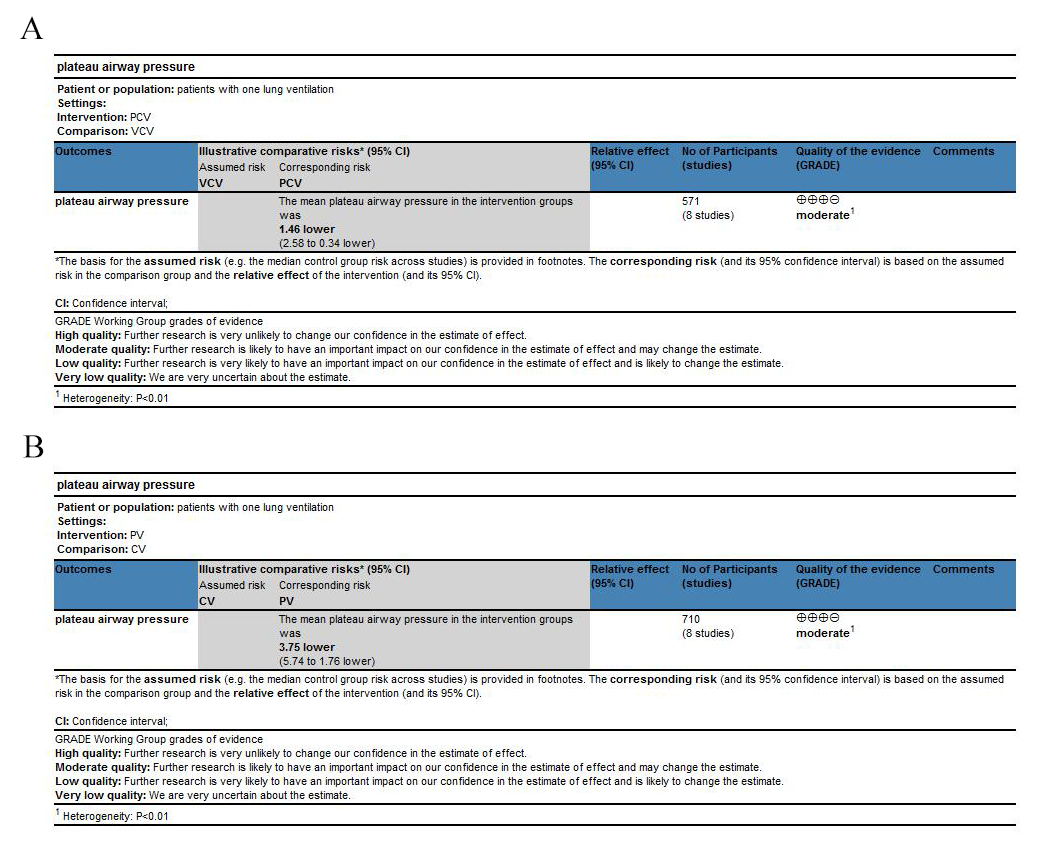


1. pressure-controlled ventilation versus volume-controlled ventilation (B) protective ventilation versus conventional ventilation
2. PaO_2_/FiO_2_


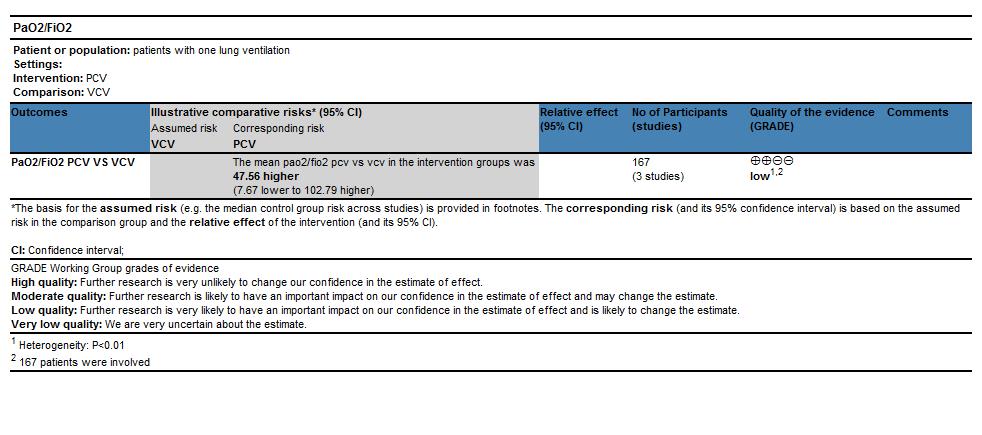


1. Mean arterial pressure


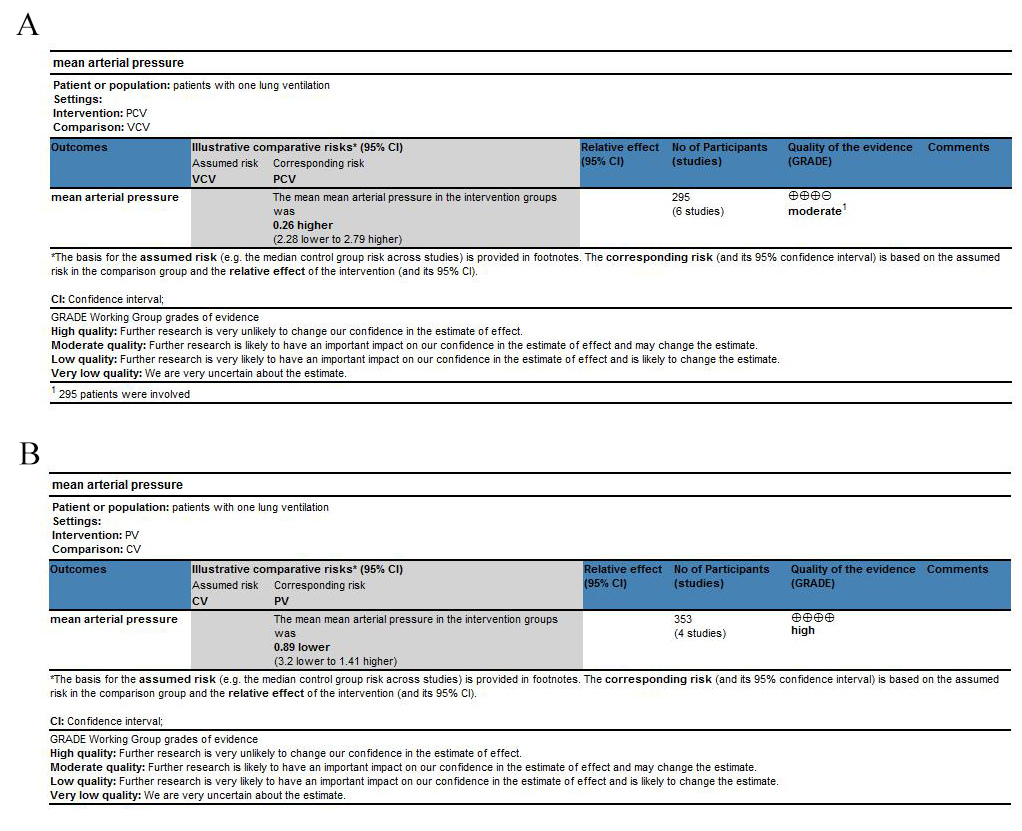


1. pressure-controlled ventilation versus volume-controlled ventilation (B) protective ventilation versus conventional ventilation

**Appendix A – Mdeline search strategy**

We developed a search strategy for Ovid Medline (1946-July 2015) and translated for the following additional database: Ovid MEDLINE(R) In-Process & Other Non-Indexed Citations (July 19, 2015); PubMed, EMBASE, the Cochrane Library.

The Medline search strategy is as follows:

1. "randomized controlled trial".pt.

2. (random$ or placebo$ or single blind$ or double blind$ or triple blind$).ti,ab.

3. (retraction of publication or retracted publication).pt.

4. or/1-3

5. (animals not humans).sh.

6. ((comment or editorial or meta-analysis or practice-guideline or review or letter or journal correspondence) not "randomized controlled trial").pt.

7. (random sampl$ or random digit$ or random effect$ or random survey or random regression).ti,ab. not "randomized controlled trial".pt.

8. 4 not (5 or 6 or 7)

9. one lung ventilation.ab,ti.

10. (perioperative care or preoperative care or postoperative care or intra-operative care or surgery or surgical or operative or surgical operations).ab,ti.

11. anesthesia.ab,ti.

12. anaesthesia.ab,ti.

13. 10 or 11 or 12

14. Single-Lung Ventilation.ab,ti.

15. Single-Lung Ventilations.ab,ti.

16. Lung Separation Techniques.ab,ti.

17. Lung Separation Technique.ab,ti.

18. 9 or 14 or 15 or 16 or 17

19. 8 and 13 and 18
